# Age differences in knowledge, attitudes and preventive practices during the COVID-19 pandemic in Spain

**DOI:** 10.1038/s41598-022-25353-5

**Published:** 2022-12-02

**Authors:** Antonio González-Herrera, Carmen Rodríguez-Blázquez, María Romay-Barja, María Falcon-Romero, Alba Ayala, María João Forjaz

**Affiliations:** 1 National School of Public Health, Carlos III Health Institute, Madrid, Spain; 2 National Epidemiology Centre, Carlos III Health Institute, Madrid, Spain; 3 National Centre of Tropical Medicine, Carlos III Health Institute, Madrid, Spain; 4 Legal Medicine, Department of Sociosanitary Sciences, University of Murcia, Murcia, Spain; 5 Department of Statistics, Carlos III University of Madrid, Madrid, Spain

**Keywords:** Human behaviour, Psychology and behaviour

## Abstract

This study aims at describing the evolution of Spanish population preventive practices during the COVID-19 pandemic of the between January and June 2021, and differences by age group. Data was drawn from the COSMO-Spain online survey, rounds (R) 4, 5 and 6. Multiple linear regression models with preventive practices as dependent variable were performed. Preventive practices (p = 0.001) and concern about coronavirus (p = 0.003) decreased throughout the three rounds, knowledge decreased from R4 to R6 (p = 0.002) and health literacy had a higher value in R6 (p < 0.001). Older the age was associated with higher the frequency of preventive practices, and levels of health literacy and concern about coronavirus (p < 0.001). The regression model showed that, in the 18–29 year group, a greater frequency of preventive practices was associated with being female (β = 0.20; p < 0.001), greater concern about coronavirus (β = 0.16; p < 0.018) and frequency of information seeking (β = 0.24; p < 0.001). For 61 years old and older, a higher frequency of preventive practices was associated with greater concern about coronavirus (β = 0.21; p < 0.002) and lower pandemic fatigue (β = − 0.13; p < 0.037). These findings point to the need for effective public health interventions tailored to the characteristics of age population groups.

## Introduction

The COVID-19 pandemic has been impacting the health of the world's population since the early 2020s. In the absence of any effective treatment, preventive measures to avoid transmission of the virus are of central importance^1^. It is therefore important to understand not only the degree of compliance with these measures, but also the factors that determine them; the aim being to ascertain the reasons behind greater or lesser degrees of compliance in order to design and prioritise effective public health interventions^2^. This was one of the reasons why in March 2020 the World Health Organisation (WHO) proposed the implementation of COVID-19 Snapshot Monitoring (COSMO), a survey that would be conducted periodically to obtain useful information for pandemic control, adapted to each epidemiological and social situation^3^. These approaches are inspired by the approach known as Behavioural Insights (BI)^4^.

Previous studies have already underscored the importance of the highlighted factors (knowledge, attitudes and preventive practices), as well as risk perception or concerns about the pandemic^4^. There are also individual variables (age, sex, educational level) that also influence greater or lesser adherence to preventive measures and the degree of COVID-19 knowledge and risk perception. Among all these factors, age is a key determinant. Older people are more vulnerable to the virus that causes COVID-19, so monitoring their adherence to preventive measures and assessing their level of knowledge about coronavirus and COVID-19 and their perception of risk is particularly important in this population group^5^. On the other hand, young people represent a highly interesting population group due to their increased mobility, number of social relations and leisure activities. Although when infected, symptoms are often mild or absent, an increase in infections among young people has been observed during certain periods of the pandemic, which has posed a challenge in containing incidence rates^6^. Ascertaining their level of knowledge about COVID-19, their degree of compliance with the main preventive measures, their concerns and their perception of risk is essential for designing communication and intervention strategies in this age group.

This study aims to answer the following research questions. First, how is the evolution of preventive practices, knowledge, attitudes, concerns, risk perception, information seeking, health literacy and pandemic fatigue of the population living in Spain between January and June 2021? Second, what are the differences by age group and other associated variables?

## Methods

This study is based on the COSMO-Spain survey, which aims to monitor the knowledge, risk perceptions, preventive behaviours and confidence of the population regarding the measures adopted during the COVID-19 pandemic in Spain, using a questionnaire based on WHO Behavioural Insights (COVID-19 Snapshot Monitoring Survey)^7^. Rounds have been conducted every two months since July 2020, and this study analyses data collected in January–February (round 4), March (round 5) and May–June (round 6) 2021. A research company invited the population that met the inclusion criteria (both sexes, aged 18 years or older, residing in Spain, and being able to understand and answer questionnaires) to answer an online survey. The people who were invited and did not answer were replaced by others with the same characteristics. A sample of 3005 people was recruited, aiming for representativeness by gender, age, educational level and area of residence (with weighting in round 6 to ensure representativeness). The epidemiological situation of each round is shown in Table 1.

**Table 1:** Epidemiological situation in the different rounds.

	Round 4	Round 5	Round 6
	25 January–1 February 2021	22–26 March 2021	24 May–3 June 2021
Cumulative incidence (last 14 days)	783.25	138.63	198.6
COVID patients in hospital	29,276	7679	8605
COVID patients in ICU	4823	159	2183
Deaths (last 7 days)	1883	265	251

Source: Spanish Health Alert and Emergency Coordination Centre (CCAES). https://www.mscbs.gob.es/profesionales/saludPublica/ccayes/alertasActual/nCov/.

Round 4 data (n = 1002) was collected between 25 January and 1 February 2021. A total of 368,334 cases were detected during the previous 14 days, with a cumulative incidence of 783.25^8^. During that week, mobility restrictions and limitations on opening hours and capacity were applied in commercial establishments in different autonomous regions.

For round 5 (n = 1002) the survey was conducted from 22 to 26 March 2021. A total of 65,194 cases were detected during the previous 14 days, with a cumulative incidence of 138.63^9^. During that pre-Easter week, mobility restrictions and restrictions on opening hours and capacity in commercial establishments were maintained in different autonomous regions.

In round 6 (n = 1001) the fieldwork was carried out between 24 May and 3 June 2021. A total of 94,236 cases were detected during the previous 14 days, with a cumulative incidence of 198.60^10^. By that date, more than 28 million doses of COVID-19 vaccine had been administered. More than 19 million, 40 per cent of the population, had received at least one dose and more than 10 million, 21.6 per cent of the population, had already received the full course.

### Variables

The questionnaire collected socio-demographic information of interest: sex, age and level of education. The questionnaire included several contextual variables, such as whether they had suffered from COVID-19, the severity of the disease if they had been infected, as well as whether someone close to them had suffered from the infection and whether they had died. The main study variables were adapted in each round according to the epidemiological and social situation^3^.

*Preventive practices* were assessed by nine items asking about the frequency of behaviour such as hand washing, use of alcohol gel, safety distance, use of masks as recommended and with friends, going to gatherings with family or friends, going to crowded places, disinfecting surfaces, and ventilating enclosed spaces (response scale between 1 "never" and 5 "always").

Participants' *knowledge* of COVID-19 (ways of becoming infected, symptoms and the correct use of preventive measures) was assessed by 11 questions. Possible answers were "yes", "no" and "don't know", the latter being considered as a wrong answer for statistical analysis.

*Health literacy* was assessed with a modified and shortened version of the HLS-EU-Q scale that measures perceived difficulty in seeking, understanding, judging and applying COVID-19-related information^11^. The COVID-19 health literacy questionnaire (CHL-Q) consists of 13 items with response options ranging from 1 to 4 from "very difficult" to "very easy", plus the option "don't know", which was not used for score-calculation purposes.

*Attitudes* towards the decisions being taken in Spain to reduce the spread of the virus were assessed by asking whether they considered these measures appropriate or exaggerated. Possible responses rated the level of agreement from 1, "do not agree at all", to 5, "strongly agree".

Feelings and concerns about the coronavirus were measured by asking specifically about the speed of spread (fast-slow), feelings of depression and fear about the pandemic, ranging from "not at all" to "very much", with a 1–5 response scale.

Another section of the questionnaire addresses perceptions of risk of infection (1, "very unlikely" to 5, "very likely"), of severity if infected (1, "very mild" to 5, "very severe") and of the ability to avoid infection or self-efficacy (1, "very difficult" to 5, "very easy"). The frequency of seeking COVID-19-related information was also asked on a scale from 1 ("never") to 5 ("several times a day").

The pandemic fatigue scale is a questionnaire based on the original version by Lilleholt et al. that assesses demotivation to comply with preventive measures and information seeking^12^. The scale includes 6 items, ranging from 1 ("strongly disagree") to 5 ("strongly agree").

### Ethical aspects

All respondents were informed about the objectives of the study and gave their consent to participate. The COSMO-Spain study has been reviewed and approved by the Research Ethics Committee of the Instituto de Salud Carlos III (CEI PI 59-2020_v2). The research was performed in accordance with relevant guidelines/regulations including the Declaration of Helsinki.

### Data analysis

A descriptive analysis was conducted of the participants’ socio-demographic characteristics, as well as of the contextual variables and the main variables of interest. All of these are shown as frequencies and percentages for categorical variables or means and standard deviations for continuous variables.

For the analysis, the age was divided into groups (18–29 years, 30–44, 45–60 and 61 or more) and the level of education into low, medium–low, medium–high and high.

A bivariate analysis was performed to assess possible associations between the different rounds and age (in groups) with the main variables: preventive practices, knowledge, literacy, attitudes, concerns, risk perception, information seeking and pandemic fatigue. ANOVA and Kruskal–Wallis tests were used according to the type of distribution of the variables.

The different preventive measures were grouped using principal component analysis to obtain an index that evaluates the degree of compliance with these measures between 0 and 100^13^. The knowledge variables were used to construct an index assessing the level of knowledge from 1 to 10, using the formula ((sum of items − minimum score)/(maximum score − minimum score)) × 10. The health literacy index was calculated as taking values between 0 and 50^11^. For pandemic fatigue, the total score was obtained by summing up the items, with a maximum of 30 points, indicating a high degree of pandemic fatigue, and a minimum of 6^12^. For the rest of the variables, the value of the scale on which the questions were answered was taken.

Finally, a multiple linear regression model was used to test the degree of association between the sociodemographic, contextual and other main variables with preventive practices as the dependent variable. This model was constructed with the full sample and for the 18–29 and 61+ age groups, to look for possible factors explaining the differences in preventive practices in both populations and, ultimately, in the incidences of coronavirus infection. Both standardized betas, as an indicator of the relationship strength, and p-level are presented. Based in previous work, we hypothesized a positive association between preventive practices and older age, being a woman, higher level of education and better knowledge and higher health literacy^14–16^.

IBM SPSS version 27 statistical software was used for all these analyses. Statistical significance was set at a p-level of 0.05 for all comparisons.

## Results

The sample consists of 3005 participants (1002 in each of rounds 4 and 5, 1001 in round 6). Socio-demographic characteristics, together with other contextual variables, are shown in Table 2. The mean age is 46.47 years (SD 14.61) and the predominant level of education is high (35.4%), followed by medium–high (25.5%). Only 9.30% of the participants have been infected by coronavirus, mostly mild cases (86.3%) and 65.9% had someone close to them who had suffered from COVID-19, and in 30.8% of cases a relative or friend died.

**Table 2:** Descriptive socio-demographic variables.

Variables	Categories	n (total 3005)	%
Gender	Male	1501	50.0
	Female	1504	50.0
Age (years)	18–29	515	17.1
	30–44	879	29.3
	45–60	1006	33.5
	61 or over	604	20.1
Level of education	Low	420	14.0
	Medium–Low	756	25.2
	Medium–High	766	25.5
	High	1062	35.4
Has had Coronavirus	Yes	280	9.30
	No	2725	90.70
Severity (if you have had coronavirus)	Mild	217	86.3
	Severe	34	13.7
Someone close to you has had Coronavirus	Yes	1982	65.9
	No	1023	34.1
Someone close to you has died of Coronavirus	Yes	610	30.8
	No	1372	69.2

### Differences by round

Table 3 shows the mean of the preventive practices index, which decreased over the 3 rounds (p = 0.001). The knowledge index values decreased from round 4 to rounds 5 and 6 (p = 0.002). The scale assessing health literacy has a higher value in the last round (p < 0.001). In parallel, the cumulative incidence in round 4 was 783.25 and in rounds 5 and 6 it dropped to 138.63 and 198.60, respectively.

**Table 3:** Main variables per round.

Round	Round 4		Round 5		Round 6		Total		p
n	1002		1002		1001		3005		
	Mean	SD	Mean	SD	Mean	SD	Mean	SD	
Preventive practices (scale 1–100)	83.18	14.73	81.62	15.82	80.26	16.87	81.69	15.87	0.001*
Knowledge (scale 1–10)	9.22	1.35	9.07	1.55	8.91	1.74	9.06	1.56	0.002*
Health literacy (scale 0–50)	30.45	9.11	30.97	9.85	32.68	9.60	31.37	9.57	< 0.001**
Attitudes: appropriate measures (scale 1–5)	2.33	1.21	2.61	1.19	2.85	1.20	2.60	1.22	< 0.001*
Attitudes: exaggerated measures (scale 1–5)	1.92	1.11	2.30	1.22	2.30	1.17	2.17	1.18	< 0.001*
Concern about coronavirus (scale 1–5)	3.91	1.00	3.59	1.05	3.43	1.09	3.64	1.06	0.003*
Rate of expansion (scale 1–5)	4.60	0.72	3.95	1.00	3.40	1.18	3.98	1.10	< 0.001*
Feeling depressed (scale 1–5)	3.45	1.18	3.26	1.21	3.09	1.24	3.27	1.22	0.002*
Fear (scale 1–5)	3.45	1.17	3.21	1.18	3.16	1.25	3.27	1.21	0.414*
Risk perception: contagion (scale 1–5)	3.13	1.07	2.91	1.08	2.60	1.09	2.88	1.10	< 0.001*
Risk perception: severity (scale 1–5)	3.26	0.90	3.19	0.95	3.11	0.98	3.19	0.95	0.03*
Risk perception: self-efficacy (scale 1–5)	2.86	1.00	3.15	0.91	3.26	0.96	3.09	0.97	0.004*
Information seeking (scale 1–5)	3.24	1.06	2.94	1.03	2.85	1.08	3.01	1.07	0.059*
Pandemic fatigue (scale 6–30)	17.73	5.06	18.20	5.34	17.47	5.11	17.80	5.18	0.006**

For Health Literacy, n = 817 (round 4), 825 (round 5), 828 (round 6), 2470 (total).

*Kruskal Wallis Test. All p-values are two-sided.

**ANOVA Test. All p-values are two-sided.

Attitudes towards thinking that the measures were appropriate increased over the 3 rounds (p < 0.001). However, attitudes towards thinking that the measures have been exaggerated also increased, at least when comparing round 4 with consecutive rounds (p < 0.001).

Concern about coronavirus decreased throughout the 3 rounds (p = 0.003). Feelings of depression about the pandemic also decreased, as did feelings of fear (p = 0.003). At the same time, the perception of risk of infection decreased over the three rounds (p < 0.001), as did the perception of severity if infected (p = 0.030). The mean values of the pandemic fatigue index remained more or less constant over the 3 rounds (p < 0.006).

### Differences by age

As can be seen in Table 4, the older the age, the higher the frequency of preventive practices (p < 0.001). The lowest level of knowledge is observed in the 18–29 age group and the highest in the 45–60 age group (p < 0.001). Health literacy values are higher in the older age groups (p = 0.003).

**Table 4:** Main variables by age groups.

Age groups	18–29 years		30–44 years		45–60 years		61 years or over		Total		p
n	515		879		1006		604		3005		
	Mean	SD	Mean	SD	Mean	SD	Mean	SD	Mean	SD	
Preventive practices (scale 1–100)	73.70	18.60	80.55	15.99	84.31	14.18	85.79	12.93	81.69	15.87	< 0.001**
Knowledge (scale 1–10)	8.76	1.95	9.10	1.60	9.23	1.34	8.99	1.41	9.06	1.56	< 0.001**
Health literacy (scale 0–50)*	30.65	9.30	30.57	9.46	31.94	9.82	32.15	9.40	31.37	9.57	0.003***
Attitudes: appropriate measures (scale 1–5)	2.59	1.16	2.46	1.17	2.59	1.24	2.81	1.27	2.60	1.22	< 0.001**
Attitudes: exaggerated measures (scale 1–5)	2.32	1.21	2.22	1.21	2.09	1.15	2.12	1.15	2.17	1.18	0.001**
Concern about coronavirus (scale 1–5)	3.38	1.07	3.53	1.07	3.79	1.05	3.80	1.02	3.64	1.06	< 0.001**
Rate of expansion (scale 1–5)	3.90	1.11	3.95	1.10	4.06	1.10	3.98	1.08	3.98	1.10	0.009**
Feeling depressed (scale 1–5)	3.54	1.16	3.46	1.15	3.18	1.23	2.91	1.22	3.27	1.22	< 0.001**
Fear (scale 1–5)	3.32	1.19	3.39	1.19	3.26	1.21	3.08	1.21	3.27	1.21	< 0.001**
Risk perception: contagion (scale 1–5)	2.85	1.07	2.93	1.11	2.94	1.13	2.74	1.05	2.88	1.10	0.001**
Risk perception: severity (scale 1–5)	2.77	0.92	3.00	0.87	3.30	0.91	3.63	0.92	3.19	0.95	< 0.001**
Risk perception: self-efficacy (scale 1–5)	3.04	1.01	3.12	0.98	3.06	0.95	3.16	0.95	3.09	0.97	0.127**
Information seeking (scale 1–5)	2.76	1.01	2.89	1.04	3.10	1.10	3.24	1.04	3.01	1.07	< 0.001**
Pandemic fatigue (scale 6–30)	19.30	4.95	18.58	5.13	17.30	5.07	16.21	5.07	17.80	5.18	< 0.001***

*For the Health literacy variable the n is 396 (18–29 years), 732 (30–44 years), 838 (45–60 years), 504 (61 years and older), 2470 (total).

**Kruskal Wallis Test. All p-values are two-sided.

***ANOVA Test. All p-values are two-sided.

The age group that tended most to think that the measures had been appropriate was the over 60 years old group, while the rest of the groups showed similar values (p < 0.001). Conversely, the younger age groups tended to think that the measures had been exaggerated, and showed the highest values (p = 0.001).

Concern about coronavirus increased with age in the different groups (p < 0.001). Feelings of depression about the pandemic decreased with increasing age (p < 0.001). Perceived risk of infection increased in all groups except those over 60 years of age (p = 0.001). Perception of seriousness in case of infection increased with age (p < 0.001). Information seeking increased with age (p < 0.001). The younger the age, the higher the levels of pandemic fatigue (p < 0.001).

### Factors associated with preventive practices

As can be seen in Table 5, a higher frequency of preventive practices was associated with being female (β = 0.13; p < 0.001) and being older (β = 0.13 between 30 and 44 years and 0.20 in the 45–60 years and over 60 years groups, with respect to the younger age group; p < 0.001). The other variables that showed a positive association with preventive practices were greater knowledge (β = 0.09; p < 0.001), health literacy (β = 0.07, p = 0.002), greater frequency of information seeking (β = 0.07; p = 0.002), greater concern about coronavirus (β = 0.22; p < 0.001) and greater sensation regarding the rate of expansion (β = 0.17; p < 0.001). Higher frequency of preventive practices was associated with a lower belief that decisions made were exaggerated (β = − 0.11; p < 0.001) and lower level of pandemic fatigue (β = − 0.07; p = 0.005). In addition, people with high or upper-middle education levels reported fewer preventive behaviours than those with low education levels (respectively, β = − 0.13 and β = − 0.09; p < 0.001 and p = 0.007).

**Table 5:** Multiple linear regression with respect to preventive practices for total and by age groups.

	Total		Age groups			
	Standardised β global	p-level*	Standardised β 18–29 years	p-level*	Standardised β 61 years or over	p-level*
**Sex (ref: Male)**						
Female	0.13	< 0.001	0.20	0.000	0.10	0.095
**Age (ref: 18–29 years)**						
30–44	0.13	< 0.001				
45–60	0.20	< 0.001				
61 years or over	0.20	< 0.001				
**Level of education (ref: Low)**						
Medium–Low	− 0.03	0.357	− 0.11	0.190	− 0.04	0.584
Medium–High	− 0.09	0.007	− 0.15	0.112	− 0.05	0.461
High	− 0.13	< 0.001	− 0.21	0.047	− 0.20	0.008
**Round (ref: round 4)**						
Round 5	0.05	0.074	0.02	0.774	0.08	0.286
Round 6	0.05	0.071	− 0.02	0.743	0.03	0.670
**Someone close to you has died from coronavirus (0 = No, 1 = Yes)**	− 0.03	0.23	0.06	0.212	0.02	0.673
**Knowledge (scale 1–10)**	0.09	< 0.001	0.11	0.051	0.07	0.223
**Health literacy (scale of 0–50)**	0.07	0.002	0.03	0.590	0.05	0.373
**Attitudes: measures were exaggerated (scale 1–5)**	− 0.11	< 0.001	− 0.11	0.077	− 0.06	0.335
**Concern about coronavirus (scale 1–5)**	0.22	< 0.001	0.16	0.018	0.21	0.002
**Rate of expansion (scale 1–5)**	0.17	< 0.001	0.06	0.369	0.12	0.083
**Feeling depressed (scale 1–5)**	− 0.02	0.531	0.03	0.614	− 0.08	0.262
**Fear (scale 1–5)**	0.04	0.131	0.03	0.623	0.06	0.428
**Risk perception: severity (scale 1–5)**	− 0.01	0.542	− 0.02	0.666	− 0.01	0.889
**Information seeking (scale 1–5)**	0.07	0.002	0.24	0.000	0.02	0.678
**Pandemic fatigue (scale 6–30)**	− 0.07	0.005	− 0.10	0.063	− 0.13	0.037

*All p-values are two-sided.

In the 18–29 year-old population, higher frequency of preventive practices was associated with being female (β = 0.20; p < 0.001), greater concern about coronavirus (β = 0.16; p < 0.018) and frequency of information seeking (β = 0.24; p < 0.001). On the other hand, people with higher levels of education reported a lower frequency of preventive practices than those with lower levels of education (β = − 0.21; p < 0.047). For those aged 61 years and older, higher frequency of preventive practices was associated with greater concern about coronavirus (β = 0.21; p < 0.002) and less pandemic fatigue (β = − 0.13; p < 0.037). In this age group, the association between high educational level and lower frequency of preventive practices was maintained (β = − 0.20; p < 0.008).

## Discussion

In the absence of an effective treatment for COVID-19, attention must be paid to the non-pharmacological measures that prevent infection and the factors that determine adherence, including age as one of the key determinants. Successive rounds of COSMO-Spain are providing valuable information for understanding the situation and proposing effective interventions to control the pandemic.

In this study, it was observed that variables such as preventive practices, knowledge, information seeking and the different concern and risk perception variables (except self-efficacy) showed a tendency to decrease between January (round 4) and June (round 6) 2021.

This may be related to the sharp drop in cumulative incidence, which was very high in round 4 and fell significantly in subsequent rounds, to the tightening of mobility restrictions and limitations on opening hours and seating capacity in commercial establishments that different autonomous regions applied in the period in which round 4 was carried out, after easing the measures during the Christmas period, and to the start of the vaccination campaign^8^.

Compared to previous COSMO-Spain rounds, some trends have been reversed^17^. Certain preventive practices, as important as the use of masks or ventilation of enclosed spaces, increased from round 2 to round 3. A tendency to improve in terms of knowledge, feeling more aware of the rate at which coronavirus was expanding or feeling depressed was also observed in these first rounds^17^. However, a decrease had already been observed since the first rounds in other analysed aspects, such as the perception of risk of infection or severity. This trend was reversed in round 4 and then declined again in subsequent rounds^3,17^. In any case, as in other studies conducted in other countries, high levels of knowledge, attitudes in favour of the measures adopted and adherence to preventive practices are observed^18,19^.

Against this background, the focus can be placed on the differences observed according to age and their possible explanations. Around mid-June 2021 (after round 6), an increase in cumulative incidence began to be observed in young people, especially those aged 20–29 years. Previously, incidence in this age group was below 100 (93.8 in the second week of June 2021)^20^, reaching values above 1000 in just over a month (1058.5 in mid-July 2021)^21^. Incidence returned to values below 100 (58.5) in early September 2021^22^. This can be explained by the fact that measures were eased in the previous weeks, together with the trends observed in the rounds analysed by this study. Furthermore, our data indicate that preventive practices, knowledge, literacy, risk perception (perceived severity) and concern about the coronavirus or the rate of expansion increase with age. However, lower age groups are more likely to feel depressed because of the pandemic, probably due to the impact it has had on their daily activity and social relationships, unlike older people. In addition, pandemic fatigue is higher in younger age groups^23,24^.

These factors may explain the aforementioned increased incidence in young people. In addition, young people have asymptomatic or mild disease if infected, are less likely to adhere to preventive measures and have a lower perception of risk compared to older age groups^25^. As a result, they play a crucial role in the evolution of incidence rates in several countries, as observed in Spain during the summer of 2021^6^. As observed in the regression by age group, being young is associated with a decreased interest in learning about COVID-19, one of the consequences of pandemic fatigue^26^. Other studies have also pointed out that although this age group tends to score high on knowledge, attitudes in favour of the measures taken and preventive behaviour, these values tend to decline over time^27,28^. Moreover, according to our findings, young men show lower compliance with preventive practices than women in the same age group. Therefore information campaigns and strategies aimed specifically at this population in order to prevent infection must be devised^29^. In any case, also in this age group, the level of compliance with the main preventive measures is high and has remained so throughout the pandemic^3,30^.

In our study, we have observed that greater adherence to preventive practices in the population is related to being female, belonging to older age groups or scoring high on variables such as knowledge and literacy, as hypothesized. These results are consistent with those of other studies^14,15^. However, some studies also point to an association between a higher level of education and a higher frequency of preventive practices^16,31^, which is inversely observed in our study. On the other hand, some sources qualify this association and point out that it may be highly variable depending on the age groups defined^32,33^. Furthermore, in some studies, educational differences were evident only among older people^34^. In any case, results are ambiguous and more studies focused on the impact of educational level on preventive behaviours by age groups are necessary.

## Limitations and strengths

The main limitation of this study is the fact that the survey was conducted online. Therefore, certain population groups with internet access problems might be under-represented in the sample. However, this sample is representative of the general Spanish population in terms of gender, age, educational level and area of residence. Moreover, this is not a longitudinal study, but snapshots in independent samples, so causal inferences cannot be made. The strengths of the study are its large sample size and the periodicity with which the study was carried out, every two months, facilitating detailed analyses of trends in the variables of interest.

## Conclusions

Despite the high scores of the Spanish population in aspects such as preventive practices or knowledge, a decrease was observed throughout the rounds of the COSMO-Spain study between January and June 2021. Concern and risk perception also decreased, all in a context of a sharp fall in cumulative incidence after high initial figures.

The findings of the present study indicate that the high transmission of the virus in young people occurred in parallel with a decrease in preventive practices, knowledge, concern and risk perception in this age group. Consistently, feelings of depression or pandemic fatigue are higher than in older people. These findings point to the need for action to ultimately prevent an even greater drop in compliance with COVID-19 preventive measures, with special attention to young people and strategies specifically targeting this age group.

## Data Availability

The data that support the findings of this study are available from the corresponding author upon reasonable request.
